# Medical Opinion and Movement

**Published:** 1907-12-21

**Authors:** 


### Medical Opinion and Movement.

Mr. H. J. Robson, of Leeds, has drawn attention to an interesting phenomenon which he has observed in several cases of different morbid conditions, both of an acute and chronic nature. The phenomenon consists in a slight eversion of the lower eyelids which is made evident upon the protusion of the tongue or the depression of the lower jaw, the lids resuming their normal position when the tongue is retracted or the mouth shut. The phenomenon is apparently never present in health, but comes and goes with the onset and cure of the disease. Mr. Robson points out that the eversion of the lower eyelids is produced by a contraction of the malar fibres of the orbicularis palpebrarum, and suggests that the co-ordinate muscular contractions are due to the proximity of the nuclei of origin of the facial and hypoglossal nerves in the medulla and pons Varolii. The condition has been observed in the course of an acute illness such as influenza, and may disappear suddenly with the subsidence of the urgent symptoms. The chronic conditions in which it has occurred have been chiefly cardiac and abdominal affections. The phenomenon has apparently not been observed before; but it will doubtless now be looked for by all who have had their attention drawn to it, and further evidence as to its significance and frequency of occurrence will be highly interesting.

An important and instructive discussion took place at a recent meeting of the Cambridge Medical Society on the question of instructing teachers in the principles of hygiene. Professor Sims Woodhead gave an account of an experimental series of lectures he has been giving during the last twelve months to school teachers, at the request of the Cambridge County Education Committee, and he expressed great satisfaction at the keenness and intelligence displayed by the teachers in the subject. He was especially impressed by the capacity they evinced for applying the principles he taught them to their every-day work and by their keen appreciation of the practical work. It was pointed out, however, that while such a voluntary course can be arranged at an education centre like Cambridge for teachers already engaged in work in the schools, similar facilities are not available in other parts of the country. All those who took part in the discussion, therefore, including Professor Clifford Allbutt and Professor Howard Marsh, were emphatic on the necessity that hygiene should form a regular part of the curriculum of all institutions in which students are trained to become teachers in schools of all grades, and a resolution was adopted to this effect. Not only would the teachers be thereby qualified to impart the rudiments of hygiene to their pupils, but by the knowledge they acquired their power of training and developing the minds and bodies of the children under their care would be greatly enhanced.

In view of the considerable testimony that has been forthcoming in recent medical publications in support of the tonic effects of formic acid and the formates on the muscular and circulatory systems, the result of certain physiological investigations on the subject by Dr. A. Goodall and Miss Isabel Mitchell, B.Sc., will come as a surprise to many. They find that these compounds have a definite depressive effect on the blood pressure in rabbits, varying according to the metallic ion employed. Thus, while potassium formate has a very marked depressive effect owing to the potash effect being added to that of the formate, calcium formate was only slightly depressive, and in concentration tended to raise the pressure. Observations on the frog's heart showed that all three salts, sodium, potassium, and calcium formate, were toxic, and experiments on nerve-muscle preparations showed that the effects on skeletal muscles were similar to those on cardiac muscle. Observations were also made on four healthy individuals. Sodium formate was used, and increasing doses were given three times a day from 1 to 2 grammes for four days. Observations on blood pressure were entirely negative, likewise dynamometric and ergographic records. It is a significant fact that most of the preparations of formates which have been considerately placed upon the market for the use of the physician, in consequence of the laudation formates have received as muscular tonics, contain also strychnine and other well-established tonic drugs.

Dr. C. Budde has evolved a new method of milk-sterilisation and many advantages are claimed for it over sterilisation by heat and other methods at present in vogue. The agent used is hydrogen peroxide, but the process of purification of milk known now as Buddeisation is considerably more complicated than the addition of a little hydrogen peroxide. An endeavour is made, not only to render the milk germ-free, but also to clean it and separate from it all extraneous matter. A demonstration of the method was made at a recent meeting of the Edinburgh Medico-chirurgical Society, and Dr. Budde himself explained it in detail. The milk from the cowshed is first passed through a heater raised to a temperature of 50° C., and then through a centrifugal cleaning machine, by which all particles of dirt are removed and also a large number of adhering bacteria. From this machine the milk is conveyed to a vat provided with a water-jacket and is mixed with a little hydrogen peroxide. It is maintained that the natural germicidal power of hydrogen peroxide is increased by the "catalase" in the milk which decomposes the peroxide liberating nascent oxygen. This decomposition takes place best at a temperature of 50° C., and the milk is accordingly kept at this temperature in the vat for three hours, after which it is transferred through sterilised tubes into sterilised bottles. It is claimed that in this way the milk is thoroughly purified and at the same time undergoes no change in its composition and nutritive value. The process is now extensively used in Denmark. Unfortunately, the outlay involved in the necessary machinery and the proper supervision of the process would prohibit its use except by large dairy companies.

